# Maternal Conditions and Perinatal Characteristics Associated with Autism Spectrum Disorder and Intellectual Disability

**DOI:** 10.1371/journal.pone.0050963

**Published:** 2013-01-07

**Authors:** Amanda T. Langridge, Emma J. Glasson, Natasha Nassar, Peter Jacoby, Craig Pennell, Ronald Hagan, Jenny Bourke, Helen Leonard, Fiona J. Stanley

**Affiliations:** 1 Telethon Institute for Child Health Research, Centre for Child Health Research, University of Western Australia, Perth, Australia; 2 Perinatal Research, Kolling Institute of Medical Research, University of Sydney, New South Wales, Australia; 3 School of Women's and Infants' Health, University of Western Australia, Perth, Australia; 4 Department of Neonatology, School of Women's and Infants' Health, University of Western Australia, Perth, Australia; Hôpital Robert Debré, France

## Abstract

**Background:**

As well as being highly comorbid conditions, autism spectrum disorders (ASD) and intellectual disability (ID) share a number of clinically-relevant phenomena. This raises questions about similarities and overlap in diagnosis and aetiological pathways that may exist for both conditions.

**Aims:**

To examine maternal conditions and perinatal factors for children diagnosed with an ASD, with or without ID, and children with ID of unknown cause, compared with unaffected children.

**Methods:**

The study population comprised all live singleton births in Western Australia (WA) between January 1984 and December 1999 (N = 383,153). Univariate and multivariate multinomial logistic regression models were applied using a blocked modelling approach to assess the effect of maternal conditions, sociodemographic factors, labour and delivery characteristics and neonatal outcomes.

**Results:**

In univariate analyses mild-moderate ID was associated with pregnancy hypertension, asthma, urinary tract infection, some types of ante-partum haemorrhage, any type of preterm birth, elective C-sections, breech presentation, poor fetal growth and need for resuscitation at birth, with all factors showing an increased risk. Severe ID was positively associated with poor fetal growth and need for resuscitation, as well as any labour or delivery complication. In the multivariate analysis no maternal conditions or perinatal factors were associated with an increased risk of ASD without ID. However, pregnancy hypertension and small head circumference were associated with a reduced risk (OR = 0.64, 95% CI: 0.43, 0.94; OR = 0.58, 95% CI: 0.34, 0.96, respectively). For ASD with ID, threatened abortion before 20 weeks gestation and poor fetal growth were associated with an increased risk.

**Conclusion:**

Findings show that indicators of a poor intrauterine environment are associated with an elevated risk of ID, while for ASD, and particularly ASD without ID, the associations are much weaker. As such, these findings highlight the importance of accounting for the absence or presence of ID when examining ASD, if we are to improve our understanding of the causal pathways associated with these conditions.

## Introduction

Autism spectrum disorders (ASD) are neurodevelopmental disorders affecting between 60 and 100 per 10,000 children [1], [2], [3], [4], [5] and are characterized by communication difficulties, anomalies in social interaction and the presence of repetitive or unusual behaviours [6]. Up to 75% of children with ASDs also have Intellectual Disability (ID) [1], [7], [8], [9] with the severity of ID symptoms possibly related to the severity of ASD [8]. Prevalence rates show ASD is increasing worldwide [2], [10], [11] the reasons for which appear to be multifactorial with indications of some influence from differences in criteria, policy and local diagnostic practices [12]. In some regions that show an increase in ASD there is a concomitant decrease in ID rates suggesting that increasing ASD prevalence may be related to growing awareness and changes in diagnostic labelling, resulting in an allocation of an ASD diagnoses over an ID diagnosis [11], [13]. In this way, children who may have been historically diagnosed with ID may now be diagnosed with ASD with comorbid ID [5], [11], [14].

As well as being highly comorbid, ASD and ID share a number of other clinically-relevant phenomena. Both may present with developmental delay, abnormal language and social difficulties [6] and are associated with medical conditions such as epilepsy, bowel dysfunction and autoimmune disorders [15], [16]. This raises questions about similarities in both diagnostic and aetiological pathways that may exist for both conditions and whether the contribution of genetics and the timing of environmental exposures overlap. It also questions whether ASD and ID should be placed on a single continuum or whether certain subgroups can be categorised as distinct disorders [17].

The aetiologies for most ASD and a large proportion of ID are unknown although in both disorders there is a recognised role of underlying genetic influence. A recent review reported that 10–20% of individuals with an ASD may have an identified genetic anomaly, but all of these also have been found among individuals diagnosed with ID, and therefore the role of these anomalies are likely to be dependent on the presence or combination of other genetic characteristics, gene expression or environmental influences [17]. Regardless, it does suggest that at least some subgroups of ASD and ID may be on the same continuum, sharing common aetiologies which may be targets for intervention. The mechanisms of genetic influence are currently ill-defined with low predictive value. This demonstrates a need to further explore common genetic bases according to phenotype, pre-diagnosis risk factors and environmental contributions, both pre and postnatal [18].

There has been substantial research investigating perinatal factors such as maternal conditions and prenatal factors associated with ASD, albeit with considerable variation in results due to aspects of methodology, diagnostic practices and case selection. Some of the more consistently reported risk factors occurring in the prenatal period are threatened abortion [19], maternal diabetes [19], [20], [21] and preeclampsia [21], [22], [23]; and for the perinatal period, preterm birth [24], [25], [26], [27], [28], low birth weight [19], [24], [29], [30], intra-uterine growth retardation [19], [22], [24], [30] and low Apgar scores [24], [29], [30], [31]. Despite suggestions of ASD occurring after “suboptimal pregnancies”, a recent meta-analysis found it difficult to conclusively identify any specific perinatal or neonatal factor/s that increases the risk of ASD [32]. It may be that the pattern of pregnancy experience either reflects an increased genetic risk, or acts with genetic vulnerability to increase a child's risk of developing ASD and that perinatal risk factors are relatively unimportant [33]. Another possibility is that the pattern of complications in conjunction with another prenatal environmental exposure may place a child on a trajectory towards developing ASD [20], [32]. There are significant methodological challenges in identifying these aetiological pathways and it is likely that there are several pathways that result in ASD.

Much less research has investigated the association of perinatal factors with ID and its subtypes, and interpretations have been further limited by a lack of diagnostic homogeneity amongst ID groups and a focus on only a few factors [34], [35]. The most common findings, however, include a positive association with maternal smoking [36], [37], maternal alcohol use [37], [38], poor fetal growth [39], [40], intrauterine infections [41], [42], asphyxia [41], [43], low birthweight [34], [39], [44], and low Apgar scores [39], [45]. As these factors are all strongly related to lower socioeconomic status, the pathways to ID reflect social pathology more clearly than those to ASD which may be more reflective of biological and genetic influences, or that those with low socioeconomic status (SES) do not seek care and are underrepresented in ASD studies.

Our previous work has shown different sociodemographic risk and intrauterine growth profiles for infants with ID alone compared to those with ASD alone or ASD with ID [4], [40]. Few studies have investigated whether maternal morbidity in pregnancy and other perinatal factors might also differ, which if demonstrated could suggest unique and different causal pathways between ASD and ID of unknown cause. The use of accurately recorded, specific perinatal events by time period in a population-based sample of ID and ASD in which social and other risk factors are controlled will help to further differentiate these pathways.

In light of evidence supporting diagnostic substitution that could be occurring [46], the observed similarities in demographic and developmental courses and the absence of confirmed aetiological pathways stemming from pregnancy exposure, we wanted to study how antenatal and perinatal characteristics compare between children diagnosed with ID, those diagnosed with ASD and those diagnosed with both conditions. The analysis requires differentiation of the type and severity of ID and whether the ID is attributable to a biomedical (e.g. Down Syndrome) or an unknown cause. It is the children without a known cause for their ID that are of particular interest to compare with children with ASD because of the observed overlap in clinical symptoms, epidemiological similarities and differences and because the aetiologies of both are unknown.

Using total population data sets of children diagnosed with ID and ASD in Western Australia (WA), our research compares the perinatal factors associated with each condition and their subgroups individually using data collected at the time of birth. Our aim was to examine maternal conditions and perinatal factors for all WA children subsequently diagnosed with ASD, with or without ID, and children with varying severity of ID, and compare findings to the rest of the birth cohort of unaffected children.

## Methods

### Ethics

Ethics approval for this study was obtained from the WA Department of Health Human Research Ethics Committee (2005/15) and the Human Research Ethics Committee at The University of WA (RA/4/1/1250).

### Study population and data sources

The study population comprised all live singleton births in WA between January 1984 and December 1999 (N = 383,153). As described elsewhere [5], the WA Data Linkage System was used to link individual records on all births in WA over this period with specific data from ASD and ID population-based disability databases. The resulting dataset was de-identified prior to being provided to the researchers such that patient consent was not required [47]. Cases were all children with a diagnosis of ASD, ID or both; controls were all children without a diagnosis and who were still alive in December 2005 so that enough time had passed to allow a diagnosis of either condition to be made by six years of age.

Data were obtained from the Midwives' Notification System (MNS), the Registrar General's birth and death registrations and several ID and ASD data sources. The MNS is a statutory collection of information recorded at the time of birth (defined as >20 weeks gestation and/or >400 g birthweight), with complete birth information from 1980 onwards [48], and includes demographic information about the mother, her pregnancy (e.g. complications and medical conditions), labour, delivery, and the infant (e.g. gender and birthweight). Birth registrations are also statutory and include additional information about parental country of birth, occupation, age and ethnicity.

The ascertainment of all children diagnosed with ASD in WA including the data sources, diagnostic procedures and service provision arrangements over this time period has previously been reported in detail [5]. However, in brief, this involved the verification of diagnoses by a Central Diagnostic Panel from 1991 and the introduction of cross-disciplinary reporting protocols in 1997 such that diagnosis could be affirmed in a more standardised and rigorous way [5]. The primary diagnostic tool used up to the mid-1990s was the DSM-IIIR [49]. In 1994 this was superseded by the DSM-IV criteria [50] and in 2000 by the DSM-IV-TR [51]. Children with ASD were identified from three overlapping sources: 1) the Disability Services Commission of WA (the government agency that is the primary service provider and assessment agency for children with ASD and/or ID); 2) the WA Register of Autism Spectrum Disorders, a prospective surveillance system of newly diagnosed cases since 1999 [52]; and 3) a retrospective dataset based on a comprehensive audit and individual case note review of all ASD cases born in WA between 1984 and 1995 and diagnosed by 1999 [5]. Children with ID were identified from the Intellectual Disability Exploring Answers Database, a WA population-based register of children with ID [53]. ID diagnostic codes are assigned by physicians using the American Association on Mental Retardation classification system [54] and, as done previously [41], [55], categorised as biomedical or otherwise. All cases with a biomedical cause (e.g. Down syndrome, Rett syndrome, etc) were excluded.

Of the 383,153 WA-born children, 869 with a biomedical cause of ID were excluded, 1,179 children were identified with an ASD: 70.1% with childhood autism (i.e. Autistic disorder), 18.4% with Pervasive Developmental Disorder not otherwise specified (PDD-NOS), 5.4% with Asperger's syndrome and in 6.1% the classification was not specified into an ASD subcategory. In 38.3% of the children with an ASD diagnosis, there was no identified ID (ASD without ID) and in the remainder, ID had been identified or, at least, not excluded (ASD with ID). A total of 4,576 children were diagnosed with an ID with no biomedical cause and with no ASD, of whom 5.2% had severe and 91.0% mild-moderate ID. The ID level was unspecified for 3.8% of children so these were included in the mild category. Thus by exclusion, the population of unaffected children in the study was 376,529.

### Analyses

Analyses were conducted in a blocked modelling approach, with blocks based on sociodemographic characteristics, maternal conditions and pregnancy complications, labour and delivery factors and neonatal outcomes. Univariate and multivariate multinomial logistic regression models were applied. The first multivariate model examined maternal conditions and pregnancy complications, which included maternal diabetes (gestational diabetes and pre-existing diabetes mellitus), threatened abortion (<20 weeks gestation), pregnancy hypertension (preeclampsia and essential hypertension), asthma, urinary tract infection during pregnancy, placenta praevia, placenta abruption and other antepartum haemorrhage (APH).

The second model, incorporated sociodemographic characteristics. These included parity (1^st^, 2^nd^, 3^rd^ or 4^th^ or more births), maternal and paternal age group (less than 20 years, 20–24 years, 25–29 years, 30–34 years, 35–39 years, 40 years and over), maternal ethnicity (Caucasian, Aboriginal, Asian and other), community-level socioeconomic status as measured by the Index of Economic Resources (IER) [56] and community accessibility/remoteness as measured by the Accessibility/Remoteness Index of Australia (ARIA) [57]. The IER is an index created by the Australian Bureau of Statistics (ABS) following each census, and is based on maternal household collection district at the time of the child's birth. Collection Districts are the smallest available area unit for which socioeconomic data can be obtained and contains approximately 200 households, although density may vary in more sparsely populated areas [58]. ARIA is also produced by the ABS and was used to assign a level of geographical remoteness (Major city, inner regional and outer regional or remote); ARIA is based on the postcode of maternal household at the time of birth.

The third model examined labour and delivery factors including type of preterm birth (spontaneous or pre-labour rupture of membranes [PROM], indicated vs. term birth), mode of delivery (spontaneous, forceps, vacuum extraction, elective caesarean section [CS], emergency CS), breech presentation and any complication of labour and delivery (e.g. precipitate delivery, fetal distress, failure to progress). The fourth model included the labour and delivery factors, as well as the sociodemographic characteristics.

The fifth model examined neonatal outcomes which included infant gender, resuscitation required at birth, percentage of optimal birthweight [59] (POBW; <75%, 75 to <85%, 85 to <95%, 95 to <105%, 105 to <115%, 115 to <125% and ≥125%) and percentage of optimal head circumference [59] (POHC; <95%, 95 to <105%, ≥105%). POBW is the ratio of the observed birth weight to optimal birth weight taking into account sex, parity, gestational age and maternal height. Similarly, POHC is the ratio of the observed to optimal head circumference. The sixth model examined neonatal outcomes and included sociodemographic characteristics.

The final model included maternal conditions and pregnancy complications, labour and delivery factors, neonatal outcomes and sociodemographic characteristics. Birth year was included in all multivariate models to allow for increases in diagnostic prevalence over time. The percentage of missing data was 5.5% for paternal age, 10.44% for the IER and 1.58% for ARIA. Odds ratios and 95% confidence intervals were calculated separately for each case group; and STATA was used for all analyses [60].

## Results

We report here on the maternal conditions and perinatal characteristics of the 1179 cases of ASD (categorised according to association with ID: ASD with ID, N = 727; ASD without ID, N = 452) and the 4576 cases of ID without ASD (categorised by level of ID: mild, N = 4339; severe, N = 237), compared with the remainder of the children born in WA between 1984 and 1999, alive in 2005 and not identified as having ID or an ASD (N = 376,539).

### Univariate analyses (Table 1)

#### Maternal conditions and pregnancy complications

**Table 1:** Univariate multinomial logistic regression.

	Mild-moderate ID (N = 4339)	Severe ID (N = 237)	ASD with ID (N = 727)	ASD without ID (N = 452)
**Block 1 – Maternal conditions and pregnancy complications**				
Maternal diabetes	1.35 (1.11, 1.63)	0.93 (0.35, 2.50)	1.38 (0.86, 2.20)	1.35 (0.74, 2.46)
Threatened abortion	0.80 (0.69, 0.93)	0.91 (0.50, 1.67)	1.60 (1.22, 2.09)	1.53 (1.08, 2.17)
Pregnancy hypertension	1.49 (1.36, 1.63)	1.41 (0.96, 2.06)	1.34 (1.07, 1.67)	0.81 (0.58, 1.15)
Asthma	1.46 (1.30, 1.63)	1.29 (0.78, 2.15)	1.18 (0.87, 1.59)	1.78 (1.29, 2.46)
Urinary Tract Infection	1.83 (1.64, 2.04)	1.41 (0.83, 2.37)	0.99 (0.70, 1.40)	1.12 (0.73, 1.70)
Placenta Praevia	1.41 (1.03, 1.92)	0.64 (0.09, 4.57)	1.05 (0.43, 2.52)	1.01 (0.32, 3.14)
Placenta Abruptio	1.68 (1.24, 2.26)	2.81 (1.04, 7.55)	1.36 (0.61, 3.05)	2.57 (1.22, 5.44)
Other APH	1.64 (1.41, 1.91)	0.83 (0.34, 2.00)	1.20 (0.78, 1.83)	0.96 (0.53, 1.74)
**Block 2 – Labour and Delivery**				
Preterm type				
*Spontaneous/PROM*	2.16 (1.93, 2.41)	2.05 (1.25, 3.36)	0.94 (0.64, 1.39)	1.06 (0.66, 1.70)
*Medically indicated*	2.16 (1.87, 2.50)	3.46 (2.11, 5.68)	1.53 (1.02, 2.28)	1.79 (1.12, 2.87)
Mode of delivery				
*Forceps successful*	0.65 (0.58, 0.74)	0.71 (0.41, 1.23)	1.22 (0.95, 1.57)	1.45 (1.06, 1.99)
*Vacuum successful*	0.79 (0.70, 0.89)	0.62 (0.34, 1.14)	0.99 (0.74, 1.32)	1.41 (1.01, 1.96)
*Elective CS*	1.07 (0.97, 1.18)	1.42 (0.97, 2.09)	1.32 (1.05, 1.66)	1.76 (1.34, 2.32)
*Emergency CS*	1.25 (1.14, 1.38)	1.79 (1.24, 2.60)	1.51 (1.20, 1.90)	1.91 (1.44, 2.53)
*Breech*	1.55 (1.36, 1.77)	1.53 (0.88, 2.68)	1.06 (0.72, 1.54)	1.68 (1.14, 2.48)
Any complication of labour or delivery	1.36 (1.28, 1.45)	1.66 (1.27, 2.17)	1.10 (0.95, 1.27)	1.54 (1.27, 1.87)
**Block 3 – neonatal outcomes**				
POBW				
*<75*	3.34 (2.92, 3.83)	6.31 (4.04, 9.85)	1.71 (1.14, 2.57)	1.06 (0.56, 2.02)
*75 to <85*	2.02 (1.84, 2.22)	1.84 (1.22, 2.78)	1.06 (0.81, 1.39)	0.94 (0.65, 1.35)
*85 to <95*	1.33 (1.23, 1.45)	1.22 (0.86, 1.73)	1.23 (1.02, 1.49)	1.16 (0.91, 1.47)
*105 to <115*	0.95 (0.86, 1.05)	0.84 (0.55, 1.29)	1.05 (0.85, 1.30)	1.17 (0.90, 1.53)
*115 to <125*	1.04 (0.91, 1.20)	0.71 (0.35, 1.42)	0.94 (0.67, 1.31)	1.22 (0.83, 1.79)
*125+*	1.59 (1.32, 1.90)	1.52 (0.70, 3.32)	1.27 (0.80, 2.00)	1.53 (0.90, 2.61)
POHC				
*<95*	1.59 (1.41, 1.79)	3.53 (2.34, 5.33)	1.13 (0.85, 1.50)	0.74 (0.48, 1.14)
*105+*	1.12 (1.05, 1.19)	1.52 (1.14, 2.01)	0.58 (0.50, 0.68)	0.62 (0.51, 0.75)
Resuscitation required	1.52 (1.43, 1.61)	2.46 (1.90, 3.18)	1.22 (1.05, 1.41)	1.22 (1.01, 1.48)
Male infant	1.61 (1.52, 1.71)	1.71 (1.31, 2.23)	4.48 (3.70, 5.41)	6.63 (5.02, 8.75)

Maternal diabetes during pregnancy (gestational diabetes or pre-existing diabetes), urinary tract infections, placenta praevia and other APH (e.g. Antepartum haemorrhage with coagulation defect, unspecified antepartum haemorrhage) were all associated with an increased risk of mild-moderate ID. Threatened abortion before 20 weeks gestation was associated with a reduced risk of mild-moderate ID, but an increased risk of ASD with and without ID. Pregnancy hypertension (preeclampsia or essential hypertension) was associated with an increased risk of mild-moderate ID and ASD with ID, while asthma was associated with an increased risk of mild-moderate ID and ASD without ID. Placenta abruptio was associated with an increased risk of mild-moderate ID, severe ID and ASD without ID.

#### Labour and delivery factors

Compared to a term birth, spontaneous preterm delivery, or preterm delivery due to premature rupture of membranes (PROM), were associated with an increased risk of mild-moderate ID and severe ID, while medically indicated preterm delivery was associated with an increased risk of all conditions. In regards to mode of delivery, compared to a spontaneous vaginal birth, an increased risk of mild-moderate ID, severe ID and ASD with ID was observed for emergency CS and any complication of labour or delivery, while breech presentation was also associated with an increased risk of mild-moderate ID. An increased risk of ASD without ID was associated with all modes of delivery, breech presentation and any complication of labour and delivery.

#### Neonatal outcomes

Findings revealed a U-shaped relationship between POBW and both mild and severe ID. A child with a POBW of less than 85% was between two to three times as likely to be diagnosed with mild-moderate ID than a child with a POBW of 95% to 104%, but the likelihood of severe ID was more than six times higher. The association between ASD and POBW was less clear, though a POBW of less than 75% and a POBW between 85% and 94% were associated with a slight increased risk of ASD with ID, while there was no association between POBW and ASD without ID.

A POHC less than 95%, or greater than 105% was associated with an increased risk of mild and severe ID, with a POHC of less than 95%, compared to a POHC of 95% to 104%, increasing the risk of severe ID by more than three and a half times. For ASD, with and without ID, compared to a POHC of 95% to 104%, a POHC greater than 105% was associated with a reduced risk. Neonatal resuscitation was markedly increased in ID but appeared relatively unimportant in ASD with or without ID. As expected, male gender was slightly increased in ID but was a major risk factor for ASD with or without ID.

### Multivariate analyses (Tables 2, 3, 4, and 5)

#### Maternal conditions and pregnancy complications

**Table 2:** Maternal conditions and pregnancy complications associated with ASD and ID^a^.

	Mild-moderate ID (N = 4339)	Severe ID (N = 237)	ASD with ID (N = 727)	ASD without ID (N = 452)
Maternal diabetes	1.27 (1.00, 1.61)	1.25 (0.46, 3.39)	1.03 (0.62, 1.69)	1.30 (0.69, 2.45)
Threatened abortion	0.86 (0.73, 1.02)	1.06 (0.56, 2.01)	1.46 (1.09, 1.96)	1.27 (0.87, 1.85)
Pregnancy hypertension	1.54 (1.40, 1.71)	1.35 (0.87, 2.08)	1.34 (1.05, 1.71)	0.73 (0.50, 1.07)
Asthma	1.32 (1.16, 1.51)	1.35 (0.76, 2.38)	0.99 (0.71, 1.38)	1.44 (1.00, 2.07)
Urinary Tract Infection	1.38 (1.20, 1.57)	1.48 (0.84, 2.62)	1.03 (0.69, 1.54)	0.93 (0.56, 1.57)
Placenta Praevia	1.64 (1.16, 2.31)	0.77 (0.11, 5.51)	0.87 (0.32, 2.33)	1.07 (0.34, 3.34)
Placenta Abruptio	1.65 (1.17, 2.31)	2.53 (0.81, 7.93)	1.76 (0.78, 3.94)	2.71 (1.21, 6.10)
Other APH	1.62 (1.37, 1.92)	0.99 (0.41, 2.41)	1.13 (0.72, 1.79)	0.90 (0.48, 1.69)

a adjusted for birth year and sociodemographics.

**Table 3:** Labour and Delivery factors associated with ASD and ID^a^.

	Mild-moderate ID (N = 4339)	Severe ID (N = 237)	ASD with ID (N = 727)	ASD without ID (N = 452)
Preterm type				
*Spontaneous/PROM*	1.85 (1.62, 2.12)	1.79 (1.01, 3.17)	0.91 (0.58, 1.42)	1.07 (0.64, 1.81)
*Medically indicated*	1.83 (1.54, 2.17)	2.36 (1.28, 4.33)	1.35 (0.87, 2.09)	1.40 (0.84, 2.33)
Mode of delivery				
*Forceps successful*	0.85 (0.73, 0.99)	0.66 (0.35, 1.25)	0.91 (0.67, 1.23)	1.10 (0.77, 1.56)
*Vacuum successful*	0.96 (0.83, 1.10)	0.52 (0.26, 1.04)	1.10 (0.80, 1.51)	1.23 (0.85, 1.79)
*Elective CS*	1.15 (1.01, 1.30)	1.13 (0.70, 1.82)	1.19 (0.91, 1.57)	1.44 (1.03, 2.02)
*Emergency CS*	1.14 (1.00, 1.29)	1.23 (0.77, 1.97)	1.33 (1.00, 1.76)	1.47 (1.05, 2.06)
*Breech presentation*	1.39 (1.18, 1.63)	0.98 (0.50, 1.93)	0.83 (0.54, 1.29)	1.18 (0.76, 1.84)
Any complication of labour or delivery	1.18 (1.09, 1.28)	1.68 (1.21, 2.33)	1.00 (0.83, 1.20)	1.15 (0.91, 1.47)

**Table 4:** Neonatal outcomes associated with ASD and ID^a^.

	Mild-moderate ID (N = 4339)	Severe ID (N = 237)	ASD with ID (N = 727)	ASD without ID (N = 452)
POBW				
*<75*	2.67 (2.26, 3.14)	4.38 (2.55, 7.53)	2.00 (1.27, 3.15)	1.69 (0.87, 3.28)
*75 to <85*	1.87 (1.67, 2.09)	1.65 (1.02, 2.65)	1.12 (0.83, 1.51)	1.08 (0.72, 1.60)
*85 to <95*	1.26 (1.15, 1.38)	1.25 (0.85, 1.85)	1.12 (0.91, 1.38)	1.19 (0.91, 1.54)
*105 to <115*	0.98 (0.88, 1.09)	0.96 (0.61, 1.53)	1.03 (0.82, 1.29)	0.99 (0.75, 1.32)
*115 to <125*	1.08 (0.92, 1.27)	0.76 (0.35, 1.61)	0.85 (0.59, 1.23)	1.09 (0.73, 1.63)
*125+*	1.33 (1.06, 1.67)	1.73 (0.77, 3.90)	1.07 (0.63, 1.84)	1.26 (0.69, 2.32)
POHC				
*<95*	1.18 (1.03, 1.36)	2.20 (1.33, 3.63)	0.98 (0.71, 1.35)	0.56 (0.34, 0.93)
*105+*	1.03 (0.91, 1.16)	1.24 (0.72, 2.14)	1.14 (0.90, 1.44)	1.29 (0.98, 1.70)
Resuscitation required	1.56 (1.45, 1.67)	2.44 (1.82, 3.25)	0.99 (0.84, 1.17)	1.24 (1.01, 1.53)
Male infant	1.62 (1.51, 1.73)	1.66 (1.24, 2.23)	4.47 (3.64, 5.49)	6.59 (4.90, 8.86)

**Table 5:** Maternal conditions, pregnancy complications, labour and delivery factors and neonatal outcomes associated with ASD and ID, adjusted for birth year and sociodemographic characteristics.

	Mild-moderate ID (N = 4339)	Severe ID (N = 237)	ASD with ID (N = 727)	ASD without ID (N = 452)
**Block 1 – Maternal conditions and pregnancy complications**				
Maternal diabetes	1.27 (0.99, 1.62)	1.20 (0.44, 3.28)	1.01 (0.61, 1.68)	1.20 (0.63, 2.26)
Threatened abortion	0.86 (0.73, 1.02)	1.06 (0.56, 2.02)	1.47 (1.09, 1.97)	1.26 (0.86, 1.85)
Pregnancy hypertension	1.39 (1.25, 1.54)	1.01 (0.64, 1.59)	1.25 (0.97, 1.61)	0.64 (0.43, 0.94)
Asthma	1.28 (1.12, 1.47)	1.27 (0.72, 2.25)	0.98 (0.71, 1.36)	1.41 (0.98, 2.04)
Urinary Tract Infection	1.36 (1.19, 1.55)	1.44 (0.82, 2.56)	1.03 (0.69, 1.55)	0.92 (0.55, 1.55)
Placenta Praevia	1.24 (0.88, 1.76)	0.49 (0.07, 3.55)	0.78 (0.29, 2.22)	0.80 (0.25, 2.52)
Placenta Abruptio	1.17 (0.83, 1.65)	1.47 (0.46, 4.70)	1.61 (0.71, 3.65)	2.18 (0.96, 4.99)
Other APH	1.41 (1.19, 1.68)	0.79 (0.32, 1.93)	1.12 (0.71, 1.78)	0.86 (0.46, 1.62)
**Block 2 – Labour and Delivery**				
Preterm type				
*Spontaneous/PROM*	1.63 (1.42, 1.87)	1.39 (0.78, 2.50)	0.83 (0.53, 1.31)	0.92 (0.54, 1.55)
*Medically indicated*	1.25 (1.04, 1.49)	1.37 (0.71, 2.64)	1.15 (0.73, 1.81)	1.45 (0.85, 2.48)
Mode of delivery				
*Forceps successful*	0.84 (0.72, 0.98)	0.66 (0.35, 1.24)	0.86 (0.63, 1.16)	1.00 (0.70, 1.43)
*Vacuum successful*	0.94 (0.81, 1.09)	0.52 (0.26, 1.04)	1.03 (0.75, 1.42)	1.13 (0.78, 1.64)
*Elective CS*	1.17 (1.03, 1.32)	1.15 (0.71, 1.87)	1.17 (0.89, 1.54)	1.34 (0.95, 1.89)
*Emergency CS*	1.03 (0.91, 1.17)	1.08 (0.67, 1.74)	1.20 (0.91, 1.60)	1.29 (0.91, 1.82)
*Breech*	1.33 (1.13, 1.56)	0.90 (0.46, 1.77)	0.89 (0.57, 1.37)	1.23 (0.79, 1.92)
Any complication of labour or delivery	1.07 (0.99, 1.16)	1.40 (1.00, 1.95)	0.98 (0.82, 1.18)	1.11 (0.87, 1.42)
**Block 3 – neonatal outcomes**				
POBW				
*<75*	2.33 (1.97, 2.75)	3.77 (2.15, 6.61)	1.83 (1.14, 2.92)	1.58 (0.80, 3.12)
*75 to <85*	1.79 (1.60, 2.00)	1.58 (0.98, 2.54)	1.10 (0.81, 1.48)	1.06 (0.71, 1.58)
*85 to <95*	1.24 (1.13, 1.36)	1.23 (0.83, 1.83)	1.12 (0.91, 1.38)	1.18 (0.91, 1.54)
*105 to <115*	0.97 (0.87, 1.09)	0.96 (0.60, 1.53)	1.02 (0.82, 1.29)	0.99 (0.74, 1.32)
*115 to <125*	1.06 (0.91, 1.25)	0.74 (0.35, 1.58)	0.84 (0.58, 1.21)	1.08 (0.72, 1.62)
*125+*	1.20 (0.95, 1.50)	1.57 (0.69, 3.56)	1.03 (0.60, 1.78)	1.20 (0.65, 2.22)
POHC				
*<95*	1.21 (1.05, 1.39)	2.22 (1.35, 3.68)	0.99 (0.72, 1.37)	0.58 (0.34, 0.96)
*105+*	0.98 (0.87, 1.12)	1.20 (0.69, 2.07)	1.13 (0.89, 1.43)	1.21 (0.91, 1.61)
Resuscitation required	1.41 (1.31, 1.52)	2.18 (1.61, 2.94)	0.97 (0.82, 1.15)	1.17 (0.95, 1.45)
Male infant	1.61 (1.50, 1.73)	1.67 (1.24, 2.24)	4.46 (3.63, 5.49)	6.58 (4.89, 8.85)

The pattern of results from the first multivariate model was similar to those seen in the univariate models, with the exception of ASD without ID. After adjusting for all maternal conditions and pregnancy complications, sociodemographic characteristics and birth year to account for diagnostic changes over time, the association previously seen between threatened abortion before 20 weeks gestation and an increased risk of ASD without ID no longer existed, although the odds remained elevated. Additionally, threatened abortion was no longer associated with a reduced risk of mild-moderate ID and placenta abruptio was not associated with an increased risk of severe ID. However, maternal diabetes was still associated with a 27% increased risk of mild-moderate ID and urinary tract infections, asthma and pregnancy hypertension increased the risk by more than 30%. All APH conditions, including placenta praevia, placenta abruptio and other APH increased the risk of mild-moderate ID by more than 60%.

Threatened abortion and pregnancy hypertension remained strongly associated with ASD with ID, with an elevated risk of almost 50%. Asthma was still associated with a 40% increased risk of ASD without ID, while placenta abruptio increased the risk by more than two and a half times.

#### Labour and delivery factors

After adjusting for all labour and delivery factors, as well as sociodemographic characteristics and birth year, several differences in outcome were observed. Any type of preterm birth (spontaneous labour, PROM or medically indicated) was still associated with a two-fold increased risk of mild and severe ID. Forceps and vacuum extraction during delivery were no longer associated with an increased risk of ASD without ID, while emergency CS was associated with an increased risk of ASD without ID and elective CS was associated with ASD with and without ID. Elective and emergency CS both continued to be associated with an increased risk of mild-moderate ID. Breech presentation remained a risk factor for mild-moderate ID and any complication of labour and delivery continued to be associated with mild and severe ID.

#### Neonatal outcomes

Poor fetal growth, as measured by the POBW, was still strongly associated with an increased risk for mild-moderate ID, severe ID and ASD with ID, but not with ASD alone. A POBW of less than 75% was associated with an increase of more than four times for severe ID, more than three times for mild-moderate ID and more than one and a half times for ASD with ID. A POBW of 75% to 84% was associated with an increased risk of mild and severe ID, while a POBW of 85% to 94% was associated with an increased risk of mild-moderate ID. A POBW of 125% and greater was also associated with an increased risk of mild-moderate ID. A POHC of less than 95% was associated with an increased risk of mild and severe ID, while for ASD without ID it was associated with a reduced risk. Resuscitation required at birth was associated with an increased risk of mild and severe ID, as well as ASD without ID, and male gender was strongly associated with all ASD diagnoses but less so with ID diagnoses.

#### Full adjusted model (Maternal conditions and pregnancy complications, labour and delivery factors, neonatal outcomes and sociodemographic characteristics)

Threatened abortion before 20 weeks gestation was associated with an increased risk of almost 50% for ASD with ID, while asthma, urinary tract infections and other APH were all associated with an increased risk of more than 25% of mild-moderate ID. Pregnancy hypertension was also associated with an increased risk of mild-moderate ID, but a reduced risk of ASD without ID (OR = 0.64, 95% CI = 0.43, 0.94). There were no positive associations with severe ID or ASD alone.

After full adjustment, spontaneous/PROM and medically indicated preterm birth were only associated with an increased risk of mild-moderate ID, as was elective CS and breech presentation. Successful forceps delivery appeared protective of mild-moderate ID. Any complication of labour or delivery was associated with a 40% increased risk of severe ID. No associations were seen with ASD diagnoses.

A POBW of less than 75% was strongly associated with an increased risk of mild and severe ID but less so ASD with ID and not at all for ASD alone.

A POHC of less than 95% remained associated with an increased risk of mild and severe ID, and a reduced risk of ASD without ID. Resuscitation required at birth only remained associated with an increased risk for mild and severe ID, and male gender continued to be strongly associated with ASD diagnoses, and much less strongly with ID.

## Discussion

This is one of the largest reported population-based studies to examine maternal conditions and perinatal characteristics associated with a diagnosis of mild-moderate ID, severe ID, ASD with ID and ASD without ID, using cases of unknown aetiology. The results clearly indicate major differences between ASD and ID with the former having weak or no associations with pre or perinatal factors and the latter strongly associated. This, along with the strong male risk, might indicate that genetic/biological pathways which we could not study in our data may be more important in ASD and that the social and perinatal risk factors are more important for ID.

Findings suggest that maternal conditions in pregnancy such as diabetes (including diabetes mellitus and gestational diabetes), pregnancy hypertension, asthma, urinary tract infections and placental abruption are associated with increased risk of subsequent ID in children, particularly mild-moderate ID. Case numbers were small in the severe category and results may have been under-estimated due to lack of precision within the data coding, or in fact it may be that severe ID is more likely to be genetic or result from severe developmental interruptions in early pregnancy. Corresponding positive association, but attenuated results, in the full model with both spontaneous and planned preterm birth and fetal growth restriction and subsequent need for resuscitation highlights the downstream effects and impact of these conditions. Preterm delivery can occur as a result of spontaneous labour, pre-labour rupture of membranes or medical indication. Findings from our study suggest that any form of preterm birth, compared to a term birth, increases the risk of mild-moderate ID. Previous research has also shown similar findings, though primarily in the very early preterm group with a gestational age of less than 32 weeks [39], [61]. Additionally, a study that examined a slightly broader group of children with neurodevelopmental disabilities, of which intellectual disability was one subgroup, found that while preterm delivery increased the risk of neurodevelopmental disabilities, a complicated preterm delivery increased the risk by more five times [62]. A meta-analysis by Gardner et al reviewed 17 autism studies and reported that preterm birth was not associated with an increased risk of ASD [32]. Of the 17 studies, nine reported no association, seven reported an increased risk and one reported a reduced risk; though there were differences in the diagnostic criteria used across the studies, with some studies using a broader criterion. In our study, we found any complication of labour and delivery to be associated with most neurodevelopmental disabilities. However, after adjusting for sociodemographic factors and maternal conditions, there was no longer any effect except for mild-moderate ID.

It has been suggested that there are two types of preterm birth; one type characterized by the presence of infection and inflammation including preterm labour, premature pre-labour rupture of the membranes, placental abruption, and cervical insufficiency and the other type characterised by placental dysfunction, attributed to maternal-placental insufficiency and includes preeclampsia and fetal growth restriction, and associated with medically indicated preterm delivery [63]. Although the mechanism of the pathophysiology of intrauterine infection during pregnancy and intellectual disability is unclear, it has been shown that intrauterine infection and inflammatory response is associated with preterm birth, which is in turn associated with increased risk of impaired brain development and poor cognitive outcomes [64]. Furthermore, maternal or placental infection may cause reduction in blood supply to the brain and result in hypoxic-ischemic brain damage; and/or increase production of pro-inflammatory cytokines [65]. These cytokines may damage oligodendrocyte progenitor cells (that protect the myelin sheaths) or damage their maturation and thus affect brain development [66], [67].

In relation to the type of preterm births associated with those pathologies causing fetal growth restriction it is likely that the reported increased risks of elective caesarean section are related to the reason for the caesarean, rather than the procedure itself. In our study, poor fetal growth was associated with an increased risk of mild-moderate ID, severe ID and ASD with ID but not ASD without ID. Our findings also show a clear gradient effect, with the risk of ID and ASD with ID increasing with poorer fetal growth measures. Previous studies have also reported an increased risk of ID [31], [39], [40], [62]. In regards to ASD, the results have been less consistent, although a recent meta-analysis identified that being small for gestational age was significantly associated with increased risk of autism [32]. Inconsistent observations are also present in our findings where we found a positive association between ASD with ID and poor fetal growth, and no association for the children diagnosed with ASD without ID. Our results strongly suggest that poor fetal growth may be associated with the presence of ID, but not with ASD alone and highlights the importance of distinguishing between the two, potentially different, phenotypes.

Compared with average head circumference, small head circumference was associated with reduced risk of ASD and although the comparable large head circumference did not reach statistical significance, the risk was elevated. Of the few studies [30], [68], [69] investigating birth head circumference in autism, most have reported no association [30], [69]. However, our finding is consistent with a recent study reporting that, although not statistically significant, children with ASD had larger head circumferences relative to body size [69]. In contrast, small birth head circumference was found to be strongly associated with ID, with a dose-response effect in increasing risk with severity of ID. This again reflects that in some ID cases the damage to the brain may have occurred early in pregnancy or be genetic with resulting microcephaly. However, there was no association with the ASD with ID group, suggesting different aetiological pathways.

Maternal asthma was associated with mild-moderate ID, a non-significant, but elevated odds for severe ID and ASD without ID with findings consistent with a recent large study [70], [71], but not with some earlier smaller studies [63], [67]. The role of β2-adrenergic agonists, medications used for asthma but also in the prevention of pre-term labour, have been identified as a potential risk factor. However, a recent study by Croen et al assessing prenatal exposure to β2-adrenergic receptor agonists and risk of ASD did not find any evidence to support a link between these [72]. Other potential risk factors that may trigger asthma are seasonality or possible misclassification with maternal respiratory infections [71]; both linked to prenatal exposure to infection which we and others have shown are associated with adverse neurodevelopmental outcomes [73].

Previous research has reported an association between urinary tract infections and intellectual disability [74], [75], though some studies suggest this is dependent on race, with the association only identified in white infants [45], [76]. In our previous study where we examined a slightly broader category of renal and urinary conditions [70], including urinary tract infections, we also found a two-fold increase in the risk of mild-moderate ID. In contrast to our findings with asthma we only found a relationship between maternal urinary tract infections and mild-moderate ID but none with ASD.

Whilst we have previously found an association between maternal diabetes and both mild-moderate ID and ASD with ID [70], in this study the relationship was only with mild-moderate ID. In a recent meta-analysis of prenatal factors associated with autism, gestational diabetes was one of a small number of factors that were positively associated with autism risk [20]. Several of the other factors, e.g. advanced parental age, being first born and maternal immigrant status were sociodemographic factors we have also already identified in our WA cohort [19], [40]. Other than maternal medication use the only other significant prenatal risk factor from the meta-analysis was an 81% elevated risk associated with maternal bleeding during pregnancy [20]. In our study, even after full adjustment, we found an association between threatened abortion before 20 weeks gestation and ASD with ID, and although non-significant, the odds were elevated for ASD without ID.

Of interest, pregnancy hypertension, including hypertension with and without proteinuria (preeclampsia), was associated with increased risk among all children diagnosed with an ID, but was protective in children with ASD only. Previous research has provided inconsistent findings in relation to both preeclampsia and ID, and preeclampsia and ASD. While some studies have reported a positive association with ID [62], [77], [78], others have not [70], [79]. Similarly, studies examining ASD have been inconsistent, but all have reported an elevated odds [21], [23], [19], [30], [80], with, to our knowledge, no one else reporting a protective effect. Possible explanations may be that the finding is due to chance, our differentiation of ASD cases with and without ID, the involvement of an unmeasured intermediate factor [81], or the neuro-protective role of magnesium sulphate, often used to treat women with preeclampsia and proven to reduce the risk of cerebral palsy [82], another adverse neurodevelopmental outcome. Further exploratory studies are required to elucidate the explanations for this relationship.

While the aetiologies for ASD and much of ID are largely unknown, findings from this study highlight the importance of the role of factors in the prenatal environment which may differ by subgroup of ID and ASD and provide clues to the pathways of development. Our findings show that indicators of a poor intrauterine environment, such as poor fetal growth, preterm birth and poorer maternal health (measured by pregnancy hypertension, diabetes, urinary tract infections, etc.) are associated with an elevated risk of ID, while for ASD, and particularly for ASD without ID, the associations are much weaker. For ASD the most significant factors may be genetic [17], [83], [84] and other environmental exposures during pregnancy (e.g. medication use [85]), as well as sociodemographic characteristics such as maternal age and birth order [4]. Although in this study we have been unable to measure the role of genetics in the development of ASD, a recent study on the heritability of autism in twins by Hallmayer et al reported that genetic factors may contribute much less than previously thought, with environmental factors estimated to explain at least 50% of the liability [18]. These findings could be due to the larger sample size and diverse ethnicities in the Californian population in comparison to Northern European populations, on which previous twin studies have been almost exclusively based [18]. Given this, we suggest that the difference in estimates of environmental liability could, at least in part, be due to variation in the genetic susceptibility of ASD in different ethnic groups.

The primary strengths of this study are the use of population-based data on ID and ASD, and the ability to investigate ASD in relation to ID subgroup. We acknowledge the difficulty in assessing cognitive function in young children with a diagnosis of ASD and as such, not all children in our study classified as having ASD with ID had information available on cognitive assessments. Therefore, we may have incorrectly assumed the presence of ID in some children with ASD, which could result in an underestimation in the differences that we have reported. Additionally, if diagnostic criteria have changed over time it is possible that the characteristics of a more recent cohort might differ from those of an earlier cohort, such as ours, which are children born between 1984 and 1999. However, assuming they remain stable over time the addition of new cases would simply provide increased power to better demonstrate some of the relationships we have detected.

In summary, over the last few decades, there has been a substantial increase in the research investigating the aetiology of ASD, producing an extensive body of literature with many inconsistent and inconclusive findings. This has fuelled the continued debate of whether ASD's are driven by genetic susceptibility, environmental factors or a combination of both. Findings from a recent genetic review have reported that the genetic anomalies that are identified in 10–20% of ASD cases are also found in individuals with ID. These findings support the concept that ID and ASD may lie on a continuum, as opposed to being different clinical entities, and may explain why there are various ID subtypes of ASD (i.e. ASD with and without ID).

While more research is needed to understand the genetic component of ASD, our findings offer insight into the differences in maternal and pre- and perinatal factors that are associated with ID and ASD, and that we suggest may lie on the causal pathway. We have shown that indicators of poor maternal health during pregnancy and suboptimal outcomes for infants are associated with an elevated risk of ID, while for ASD the associations are fewer and weaker. Although the associations with mild-moderate ID were stronger than those with severe ID, this may be attributed to the differences in sample size.

Given our findings, it is important that future research examines ASD according to the absence or presence of ID when investigating the causal pathways associated with both of these conditions. As newer data become available, and the number of cases within each group increases, we will have the ability to re-examine some of the factors within this study that have been identified as having a borderline association with a diagnosis. Replication is warranted, especially given the continuing increase in rates of ID and ASD reported in the literature [5], [86], if we are to improve our understanding of causal pathways, engage in better prevention programs and promote appropriate interventions.
